# Predictive factors for prolonged hospital stay after retropubic radical prostatectomy in a high-volume teaching center

**DOI:** 10.1590/S1677-5538.IBJU.2017.0339

**Published:** 2018

**Authors:** Rafael F. Coelho, Mauricio D. Cordeiro, Guilherme P. Padovani, Rafael Localli, Limirio Fonseca, José Pontes, Giuliano B. Guglielmetti, Miguel Srougi, William Carlos Nahas

**Affiliations:** 1 Divisão de Urologia, Instituto do Câncer de Estado de São Paulo, Faculdade de Medicina da Universidade de São Paulo, São Paulo, Brasil

**Keywords:** Prostatectomy, Therapeutics, Retrospective Studies

## Abstract

**Objective:**

To evaluate the length hospital stay and predictors of prolonged hospitalization after RRP performed in a high-surgical volume teaching institution, and analyze the rate of unplanned visits to the office, emergency care, hospital readmissions and perioperative complications rates.

**Materials and Methods:**

Retrospective analysis of prospectively collected data in a standardized database for patients with localized prostate cancer undergoing RRP in our institution between January/2010 - January/2012.

A logistic regression model including preoperative variables was initially built in order to determine the factors that predict prolonged hospital stay before the surgical procedure; subsequently, a second model including both pre and intraoperative variables was analyzed.

**Results:**

1011 patients underwent RRP at our institution were evaluated. The median hospital stay was 2 days, and 217 (21.5%) patients had prolonged hospitalization. Predictors of prolonged hospital stay among the preoperative variables were ICC (OR. 1.40 p=0.003), age (OR 1.050 p<0.001), ASA score of 3 (OR. 3.260 p<0.001), prostate volume on USG-TR (OR, 1.005 p=0.038) and African-American race (OR 2.235 p=0.004); among intra and postoperative factors, operative time (OR 1.007 p=0.022) and the presence of any complications (OR 2.013 p=0.009) or major complications (OR 2.357 p=0.01) were also correlated independently with prolonged hospital stay. The complication rate was 14.5%.

**Conclusions:**

The independent predictors of prolonged hospitalization among preoperative variables were CCI, age, ASA score of 3, prostate volume on USG-TR and African-American race; amongst intra and postoperative factors, operative time, presence of any complications and major complications were correlated independently with prolonged hospital stay.

## INTRODUCTION

The number of new cases of prostate cancer worldwide is higher than 1.1 million each year, which represents 15.3% of all incident cancer cases in developed countries and 4.3% of the cases in developing countries (1). For patients with a clinically localized disease, a series of alternative treatment options is currently available. However, for patients with a life expectancy exceeding 10 years, radical prostatectomy (RP) continues to be one of the standard treatments (2).

After its first description in 1905 by H.H. Young (3), RP was initially associated with significant peri and postoperative morbidity. However, the technique of retropubic radical prostatectomy (RRP) was subsequently standardized by Walsh et al. (4), with significant improvement in perioperative, oncological, and functional outcomes (5). The practices of hospitalization and convalescence time post-RRP have also clearly accompanied the evolution in the refinements of the RRP technique. With improvements in the anesthetic technique and intra-operative care, the mean hospitalization time has decreased (6-8); subsequently, this time was further reduced by the establishment of optimized strategies for perioperative care after RRP, which resulted in short hospitalization, without an associated increase in the postoperative complication rates (9).

This perioperative care pathway can be further optimized by determining potentially modifiable predictive factors for prolonged hospitalization after RP. The importance in evaluating those predictors becomes even clearer when we observe that the primary definitive treatment has the highest impact in the overall costs of prostate cancer care, both in the short and long term (10, 11). Therefore, determining the factors that predict longer hospitalization after RRP may impact significantly in hospital costs, in patient management and, finally, in the governmental spending plans in public institutions involved in prostate cancer care.

However, studies evaluating predictors for prolonged hospitalization after PRR are still rare in the literature (12) and non-existent in our setting - a public high volume teaching hospital. We cannot overemphasize that the real benefits of minimally invasive RP over RRP are still unclear in the literature (13) and that in many areas of the World open RP is still the most common surgical approach to treat clinically localized prostate cancer (14).

We sought in this study to evaluate the length of hospital stay and the impact of pre, intra, and post-operative factors on the incidence of prolonged hospitalization in patients who underwent RRP in a high-volume teaching hospital.

## MATERIALS AND METHODS

### Study Design

The study was a retrospective analysis of prospectively collected data in a standardized database for patients with clinically localized prostate cancer who underwent surgical treatment in our institution. The surgical procedures were performed by residents during their last year of training under the supervision of one of the staff members; the rotation of each resident in the Urology Oncology Department lasts 5 months, and over this period each resident performs on average 300 surgical procedures, being 100 PRRs.

### Patient Selection

We included in this study all patients with clinically localized or locally advanced prostate cancer, who underwent RRP in the instituting between January 2010 and January 2012. All patients with PSA>10mg/mL and/or Gleason score ≥8 underwent preoperative bone scans, in order to excluded the presence of bone metastases. All patients included in the study signed an informed consent form authorizing data collection.

### Pre-operative Evaluation

All patients had demographic and clinical data collected and underwent a standardized pre-operative evaluation, including Digital Rectal Exam (DRE), ultrasound-guided trans-rectal biopsy (TRUS), evaluation of the serum PSA level and bone scan when necessary. Prostate magnetic resonance imaging (MRI) was done in select cases, at the discretion of the attending physician. The preoperative clinical risk stratification was done by cardiologists or general practitioners following the American Cardiology Association guidelines (16).

### Surgical Technique - RRP

RRP was done using the technique standardized by Srougi et al. (17, 18). The preferred anesthetic modality was the combination of general and epidural anesthesia. RR|P was performed through a medial infra-umbilical incision; the pre-peritoneal retropubic space was dissected to expose the anterior aspect of the prostate and the vesico-prostatic transition. When indicated, pelvic lymph node dissection (PLND) was performed prior to RP including only the obturator fossa (level I). As a rule, PLND was performed in intermediate and high risk patients according to NCCN criteria. A standard retrograde approach to RP was performed in every case including opening of the endopelvic fascia, ligation of the dorsal venous complex, retrograde dissection of the prostatic apex and neurovascular bundles, bladder neck dissection (without sparing), seminal vesicle dissection, bladder neck closure in “tennis racket” and, finally, vesicourethral anastomosis. A Penrose drain was left in place and removed before patient discharge.

### Intraoperative Data Collection

The data collected intra-operatively included: type of anesthesia, operative time, estimated blood loss (EBL) (calculated through the weighting of surgical sponges and volume of blood aspirated from the surgical field), blood transfusion, neurovascular bundle preservation (unilateral/bilateral, partial, or complete), presence or absence of a medium lobe, lymph node dissection and extension, intraoperative complications (vascular injury, rectal injury, obturator nerve injury, etc.).

### Perioperative Care and Hospital Discharge Criteria

A sole dose of an intravenous first generation cephalosporin was administered during anesthesia induction. Intermittent compression stockings were routinely used in the trans-operative period. Pharmacological prophylaxis of thromboembolic events was not done routinely.

In the immediate post-operative period (iPO) the patients were offered oral liquids freely; regular diet was offered in the morning after the surgery. Walking was also started in the first postoperative day. Analgesia was done routinely with common painkillers combined with anti-inflammatory medication intravenously in the iPO and orally in the first day after surgery. The Penrose drain was removed usually due to hospital discharge.

The patients received hospital discharge within 2 days post-operatively as long as they fulfilled the following criteria: were afebrile, no signs of orthostatic hypotension, pain controlled with oral medication, were able to walk with minimal discomfort, tolerated the diet without nausea or vomiting, did not present post-operative complications or intercurrences that required daily medical evaluation (Table-1).

**Table 1:** Peri-operative Care and Hospital Discharge Criteria.

	Immediate post-operative	First/second days PO.	Hospital Discharge Criteria
**Activity**	– Sit on the armchair 4 hours after surgery – Respiratory Physiotherapy in cases with higher risk of pulmonary complications, at the surgeon's discretion	– Walking in the morning on the first P.O. – Motor and respiratory Physiotherapy, at the surgeon's discretion	– Able to walk with minimal discomfort, being confident and comfortable to leave the hospital.
**Diet**	Liquid diet	Laxative general diet	– Tolerating general diet without nausea or vomiting
**Analgesia and medication**	– NSAIDs + common painkillers in routine (opioids orally if necessary) – Prophylactic antibiotic therapy until 24 hours after the procedure – Proton pump inhibitors	– NSAIDs + common painkillers in routine (opioids orally if necessary) – Mass forming laxatives orally – Proton pump inhibitor – Reintroduction of the usual medications	– Afebrile, without orthostatic hypotension, pain controlled with oral medication
**Care and recommendations**	– Recommendations about care with the drain, incision, collecting pouch, fall prevention No lab exams were ordered routinely	– Penrose drain removed – Recommendations about the use of the urine collector at home (leg collector offered to the patients)	Attested ability of the patient, family member, or companion to understand the guidelines about physical activity, medication, pain control, constipation prevention, care with the incision, care with the Foley, return at the clinic

### Anatomopathological Evaluation

The surgical specimens were processed according to the recommendations of the American Society of Clinical Pathologists (19). Clinical staging was done according to the TNM system (15). Histopathological findings were analyzed as potential predictors for prolonged hospitalization including prostate weight, Gleason score, pathological stage, tumor volume, PSMs and its location and number of lymph nodes removed.

### Post-operative Evaluation

The length of the hospital stay was calculated by subtracting the date of admission from the date of discharge. Patients who were re-hospitalized within 28 days after the surgery were considered “readmissions” (20). Since there is no standardized definition, neither national nor international, for prolonged hospitalization, it was defined in this study as a hospital stay longer than the upper quartile of hospitalization time in our series (>2 days).

Complications that occurred during the surgical procedure or within 90 days after the surgery were analyzed and classified according to the modified Clavien-Dindo system (21).

### Definition of Complications

Perioperative blood transfusion was generally indicated for patients with symptomatic anemia and serum hemoglobin levels < 7g/dL. For intermediary levels of hemoglobin (7-10 g/dL), blood transfusion was indicated in case of real or potential continuous hemorrhage, or in the presence of risk factors for secondary complications to insufficient oxygenation (for example, ischemic heart disease).

Paralytic ileus was defined as nausea, vomiting and/or abdominal distension post-operatively requiring hospitalization for longer than 2 days in the absence of mechanical intestinal obstruction.

Symptomatic lymphocele was defined as a pelvic fluid collection (especially along the iliac vessels) in patients who underwent lymphadenectomy associated with pelvic pain or pressure, lower limb edema, hydronephrosis, deep vein thrombosis, or infection/sepsis.

### Statistical analysis

Descriptive statistics was used initially to analyzed the frequency of the variables included in the study. Univariate analysis was performed to select the potential predictors to be included in the logistic regression model. Logistic regression was then used to determine the factors that independently predict prolonged hospitalization after RRP in our institution. A model including only preoperative variables was initially built to determine the factors that predict hospitalization time before surgery; subsequently a second model, including pre, intra, and postoperative variables were analyzed. Preoperative variables included in the model were age, race, BMI, PSA, Charlson comorbidity index (CCI), ASA score, previous abdominal surgery, clinical stage, prostate volume, biopsy (Gleason score) and percentage of positive cores, NCCN risk stratification. Intra and postoperative factors included in the analysis were: type of anesthesia, operative time, EBL, transfusion, nervesparing approach, lymph node dissection, prostate weight, tumor volume, Gleason score specimen, positive margin rates, pathologic stage and post-operative complications. For statistical analyses purposes, CCI was analyzed as a binary (0 vs. ≥1 comorbidities) and also as a continuous variable, in order to ensure that the categorization of CCI did not affect the results. The data were analyzed using Stata^®^ 13.1 software. P-values lower than 0.05 were considered statistically significant.

## RESULTS

### Clinical and demographic data and pre-operative tumor characteristics

Between January 2010 and January 2012, 1011 patients underwent a RRP in our institution and were included in the study; no patient who underwent RRP for primary treatment of a localized prostate cancer during the period was excluded from the study. However, 110 patients undergoing laparoscopic RP and 5 patients submitted to salvage RP after radiotherapy in this time frame were excluded from the study. The pre-operative clinical, demographic, pathological characteristics of the patients are presented in Table-2.

**Table 2:** Pre-operative clinical, demographic, and pathologic characteristics.

Characteristics		N=1011
**Age (years) - median (IQR)**		65.4 (60.2-69.7)
**Race (%)**		
	White	825 (81.6%)
	Black	73 (7.2%)
	Yellow	20 (2%)
	Other	93 (9.2%)
**BMI, kg/m^2^- median (IQR)**		26.6 (24.2-29.4)
**Charlson Comorbidity Index (CCI)**		
	0	738 (73%)
	≥1	273 (27%)
**CCI continuous variable - median (IQR)**		0 (0-1)
**ASA Score**		
	1	304 (30.1%)
	2	656 (64.9%)
	3	51 (5%)
**Previous abdominal surgery**		193 (19.1%)
**PSA (ng/mL) - median (IQR)**		9.1 (6-14.3)
**Prostate volume TRUS (mL) - median (IQR)**		42 (30-68.2)
**Biopsy Gleason score**		
	≤6	672 (66.5%)
	7	263 (26%)
	≥8	76 (7.5%)
**Clinical Stage**		
	T1c	539 (53.3%)
	T2	404 (40%)
	T3	68 (6.7%)
**Risk stratification - NCCN**		
	Low or very low	296 (29.3%)
	Intermediate	497 (49.1%)
	High or very high	218 (21.6%)

### Perioperative Outcomes

The most common type of anesthesia used was general anesthesia associated with epidural (92.2%), which is the standard at our institution for RRP. The median operative time was 130 minutes (IQR, 110-160); the median EBL was 600 mL (IQR, 300-1000) and 52 patients (5.14%) received blood transfusions peri-operatively. PLND was performed in 63% of patients; no patient underwent extended PLND, since it was not the standard at our institution during the period of this study.

**Figure 1:**
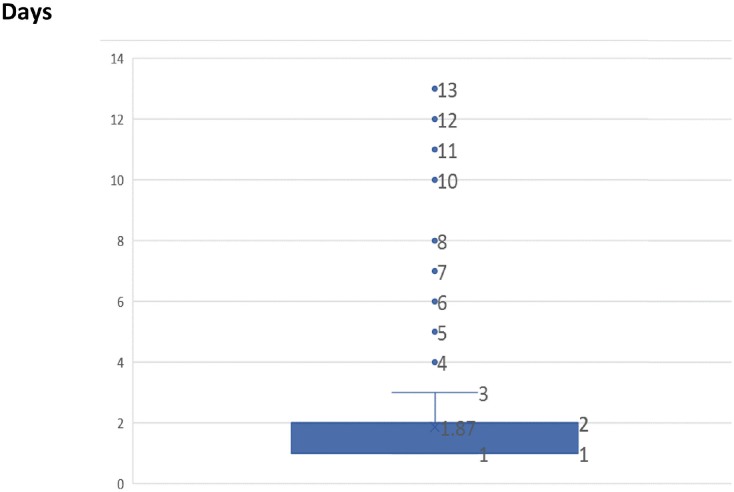
Hospitalization Time.

The median hospitalization time was 2 days (IQR, 1-2; mean 1.86±1.27 days); 217 (21.5%) patients presented prolonged hospitalization according to the definition adopted in this study (>2 days). Figure-1 shows a Box Plot graph of the hospitalization time in this study.

Hospital readmission was necessary in 28 (2.7%) patients, while 74 (7.3%) patients had non- -scheduled visits to the emergency room or clinic without need for hospitalization.

Perioperative results are shown in Table-3.

**Table 3:** Peri-operative outcomes.

Perioperative variables		N=1011
**Anesthesia**		
	General	62 (6.1%)
	Peridural + general	932 (92.2%)
	Rachianesthesia + general	17 (1.7%)
**Obturatory fossa lymphadenectomy**		
	Yes	638 (63%)
	No	373 (37%)
**Operative time (min) - median (interquartile variation)**		130 (110-160)
**Estimated bleeding (mL) - median (interquartile variation)**		600 (300-1000)
**Rate of blood transfusion**		5.14%(52/1011)
**Hospital stay - median (interquartile variation)**		2 (1-2)
	≤2 days	794 (78.5%)
	>2 days	217 (21.5%)
**Non-scheduled visits to the ER or clinic**		74 (7.3%)
**Hospital readmissions**		28 (2.7%)

### Surgical Complications

We observed 156 postoperative complications in 141 patients (14.5%). Intra-operative complications included 5 rectal injuries and one obturator nerve injury. These complications were not classified according to the Clavien system, since it applies solely to post-operative complications.

The classification of complications according to the Clavien System is presented in Table-4. There were no deaths within 90 days after surgery and/or due to the surgical procedure (Grade 5).

**Table 4:** Complications (1).

Clavien Classification	Number of Patients	Percentage of Patients, %	Incidence of Complications
I	21	14.3%	2.0%
II	66	44.9%	6.5%
IIIa	23	15.6%	2.3%
IIIb	18	12.2%	1.8%
IVa	11	7.5%	1.1%
IVb	2	1.4%	0.2%
V	0	0	0
Total	147	100	14.5% (147/1011)
Minor post-operative complications (Grades I + II) *	87	61.7%	8.5%
Major post-operative complications (Grades III + IV) *	54	38.3%	5.4%

(1) When patients presented more than one complication, the higher Clavien Score was considered.

* Excluding intra-operative complications.

### Histopathological Findings

The histopathological findings are presented in Table-5.

**Table 5:** Histopathological findings.

Histopathological findings	N=1011
**Prostate weight (g) - median (IQR)**	43 (34-68.2)
**Pathological staging**	
pT2a	153 (15.1%)
pT2b	74 (7.3%)
pT2c	524 (51.8%)
pT3a	157 (15.6%)
pT3b	103 (10.2%)
**Tumor volume (%) - median (IQR)**	15 (10-30)
**Positive surgical margin rates - overall**	269 (26.6%)
pT2	175/751(23.3%)
pT3	94/260 (36.2%)
**Positive surgical margins - location**	
Apex	74 (7.3%)
Bladder neck	11 (1%)
Circumferential	121 (12%)
Multifocal	63 (6.2%)
**Gleason Score - Specimen**	
≤6	375 (37.1%)
7	534 (52.8%)
≥8	102 (10.1%)

The median prostate weight was 43 (3468.2) g. The majority of patients (74.2%) presented organ-confined disease; 15.1% were pT2a, 7.2% were pT2b, and 51.8% were pT2c. Seminal vesicle invasion (pT3b) was identified in 10.2% of the patients and extra prostatic extension (pT3a) was found in 15.6%.

The most common Gleason score in the surgical specimen was 7, which corresponded to 52.8% of the patients (396 patients Gleason 3+4, 39.1%; 138 patients Gleason 4+3, 13.6%). The median tumor volume was 15 (10-30)%.

The overall PSM rate was 26.6%; in patients with pT2 disease the PSM rate was 23.3% while in patients pT3 disease the rate was 36.2%. The most common location of positive margins was in the prostate apex (74/1011, 7.3%).

### Predictors of prolonged hospitalization - Univariate analysis

We initially conducted a univariate analysis of predictors of prolonged hospitalization including pre, intra, and post-operative variables. In this analysis, among the pre-operative variables, the following were correlated with prolonged hospitalization: age, race, ASA score, CCI both as a binary and continuous variable, and prostate volume in the TRUS (Table-6A).

**Table 6 - A:** Univariate analysis - Predictors of prolonged hospitalization amongst pre-operative variables.

Pre-operative variables		Hospital stay		P-value
		≤2 days	>2 days	
**Age (years) - median (IQR)**		64.86 (59.59-69.36)	67.22 (63.51-71.13)	<0.001
**BMI (kg/m^2^)- median (IQR)**		26.7 (24.27-29.40)	26.40 (23.80-29.30)	0.531
**Race**				
	White	670 (84.4%)	155 (71.4%)	
	Black	45 (5.6%)	28 (13%)	<0.001
	Asian	16 (2%)	4 (1.8%)	
	Other	63 (8%)	30 (13.8%)	
**ASA score**				
	1	241 (30.8%)	53 (24.4%)	
	2	513 (65.4%)	143 (65.9%)	0.001
	3	30 (3.8%)	21 (9.7%)	
**CCI**				
	0	602 (75.8%)	136 (62.7%)	<0.001
	≥1	192 (24.2%)	81 (37.3%)	
**CCI as continuous variable - median (IQR)**		0 (0-1)	0 (0-1)	0.001
**Previous abdominal surgery**				
	Yes	158 (19.9%)	35 (16.1%)	0.248
	No	636 (80.1%)	182 (83.9%)	
**PSA (ng/mL) - median (IQR)**		9.25 (6.00-14.25)	8.55 (5.7-14.30)	0.469
**Clinical stage**				
	T1c	429 (54%)	110 (50.7%)	
	T2	313 (39.4%)	91 (42%)	0.671
	T3	52 (6.6%)	16 (7.3%)	
**Gleason Score - biopsy**				
	≤6	533 (67.1%)	139 (64%)	
	7	201 (25.3%)	62 (28.6%)	0.624
	≥8	60 (7.6%)	16 (7.4%)	
**Risk stratification-NCCN**				
	Low or very low	236 (29.7%)	60 (27.6%)	
	Intermediate	386 (48.6%)	111 (51.2%)	0.783
	High or very high	172 (21.7%)	46 (21.2%)	
**Prostate volume TRUS (mL) - median (IQR)**		40 (30 – 50)	43 (30-60)	0.006

Considering intra and post-operative variables, the following were correlated with prolonged hospitalization: EBL, blood transfusion rate, operative time, presence of post-operative complications (of any grade, minor and major complications), and prostate weight in the histopathological evaluation (Table-6B).

**Table - 6B:** Univariate analysis - Predictors of prolonged hospitalization amongst intra and post-operative variables.

Intra and post-operative variables		Hospital stay		P-value
		≤ 2 days	>2 days	
**Intra-operative factors**				
**Anesthesia**				
	General	45 (5.7%)	17 (7.8%)	
	Peridural + general	735 (92.6%)	197 (90.8%)	0.47
	Rachianesthesia + general	14 (1.7%)	3 (1.4%)	
**Estimated bleeding (mL) - median (IQR)**		600 (300-900)	700 (350-1100)	0.02
**Blood transfusion rate**				
	Yes	30 (3.8%)	22 (10.1%)	<0.001
	No	764 (96.2%)	195 (89.9%)	
**Operative time (min) - median (IQR)**		130 (110-155)	140(120-180)	0.004
**Type of preservation of the neurovascular bundle**				
	No preservation	68 (8.6%)	22 (10.2%)	
	Bilateral	629 (79.2%)	157 (72.7%)	0.104
	Unilateral	97 (12.2%)	37 (17.1%)	
**Obturator lymphadenectomy**				
	Yes	500 (63%)	138 (63.6%)	0.929
	No	294 (37%)	79 (36.4%)	
**Post-operative factors**				
**Any complication (All Clavien grades) (** **1** **)**				
Yes		95 (12%)	52 (24%)	<0.001
No		699 (88%)	165 (76%)	
**Minor complications (Clavien I and II)**				
	Yes	59 (7.4%)	28 (13%)	0.016
	No	735 (92.6%)	189 (87%)	
**Major complications (Clavien III and IV)**				
	Yes	30 (3.8%)	24 (11%)	<0.001
	No	764 (96.2%)	193 (89%)	
**Rate of positive surgical margins**				
	Overall	205 (25.8%)	64 (29.5%)	0.157
	pT2	134/591(22.7%)	41/160 (25.6%)	0.498
	pT3	71/203 (35%)	23/57 (40%)	0.555
**Pathological staging**				
	pT2a	121 (15.3%)	32 (14.7%)	
	pT2b	64 (8%)	10 (4.7%)	
	pT2c	406 (51.1%)	118 (54.4%)	0.523
	pT3a	122 (15.4%)	35 (16.1%)	
	pT3b	81 (10.2%)	22 (10.1%)	
**Specimen Gleason score**				
	≤6	312 (39.3%)	71 (32.7%)	
	7	405 (51%)	125 (57.6%)	0.342
	≥8	77 (9.7%)	21 (9.7%)	
**Tumor volume(%) median (IQR)**		16 (10-30)	15 (10-30)	0.944
**Prostate weight (g) median (IQR**)		41 (34-54)	43.5 (34-62)	0.006

1 -Included intra-operative complications, but were not classified in the Clavien system

### Predictors of prolonged hospitalization including pre-operative variables

In this first logistic regression model, only preoperative variables were included (Table-7A). CCI was initially analyzed as a binary variable (0 vs. ≥1); subsequently, we also performed a sensitivity analysis including CCI as continuous variable, to ensure that our categorization of CCI did not affect the results. The independent predictors of prolonged hospitalization in this model were age (OR 1.050, CI 95% 1.023-1.078, p<0.001), CCI as a binary (ICC ≥1 vs, ICC 0, OR. 1.818, IC95% 1.272-2.6, p=0.001) and as continuous variable (OR 1.401, CI 95% 1.118-1.756, p=0.003), TRUS prostate volume (OR 1.006, CI 95% 1.001-1.011, p=0.033) and black race (OR 1.910, CI 95% 1.103-3.307, p=0.021).

**Table - 7A:** Multivariate analysis - Predictors of prolonged hospitalization including solely pre-operative factors.

Pre-operative variables		Odds ratio	CI 95%	P value
**Age**	Continuous variable	**1.050**	**1.023 – 1.078**	**<0.001**
**CCI**	Continuous variable	**1.401**	**1.118 – 1.756**	**0.003**
**CCI (Binary)**	0	Reference	–	–
	≥1	**1.818**	**1.272 – 2.600**	**0.001**
**ASA score**	1	Reference	–	–
	2	1.175	0.791 – 1.744	0.425
	3	**3.192**	**1.616 – 6.308**	**<0.001**
**Prostate volume (TRUS) - (mL)**	Continuous variable	**1.006**	**1.001 – 1.011**	**0.033**
**Race**	White	Reference	–	–
	Black	**1.910**	**1.103 – 3.307**	**0.021**
	Asian	1.546	0.789 – 3.031	0.204
	Other	1.317	0.410 – 4.228	0.644

### Predictors of prolonged hospitalization including pre, intra, and post-operative factors combined

In the second logistic regression model we analyzed predictors of prolonged hospitalization based on pre, intra, and post-operative factors combined. Similarly to the first model, we evaluated CCI as a binary and as a continuous variable. The independent predictors of prolonged hospitalization in this model were age (OR 1.042, CI95% 1.016-1.070, p=0.002), CCI both as a binary variable (CCI >1 vs. CCI 0, OR 1.832, CI 95% 1.2772.682, p=0.001) or as a continuous variable (OR 1.461, CI 95%, 1.150-1.855, p=0.002), ASA score 3 (OR 3.192, CI 95% 1.585-6.430, p=0.001), black race (OR 1.788, CI 95% 1.037-3.083, p=0.036), operative time (OR 1.006, CI 95% 1.001-1.011, p=0.019), presence of any grade of post-operative complications (OR 1.7945, CI 95% 1.072-3.008, p=0.026) or major complications (OR 2.104, CI 95% 1.100-4.025, p=0.0125), and pathology prostate weight (OR 1.009, CI 95% 1.003-1.015, p=0.006) (Table-7B).

**Table - 7B:** Multivariate analysis - Predictors of prolonged hospitalization including combined pre, intra, and post-operative factors.

Combined pre, intra, and post-operative variables		Odds ratio	CI 95%	P value
**Age**	**Continuous variable**	1.042	1.016 – 1.070	0.002
**CCI**	**Continuous variable**	1.461	1.150 – 1.855	0.002
**CCI (binary)**	0	Reference	–	–
	≥1	1.832	1.277 – 2.628	0.001
ASA score	1	Reference	–	–
	2	1.028	0.702 1.506	0.887
	3	3.192	1.585-6.430	0.001
Race	White	Reference	–	–
	Black	1.788	1.037 – 3.083	0.036
	Asian	1.189	0.604 – 2.338	0.616
	Other	1.639	0.565 – 4.756	0.363
Estimated bleeding (mL)	Continuous variable	1.000	1.000 – 1.001	0.552
**Operative time (min)**	Continuous variable	1.006	1.001 – 1.011	0.019
Blood transfusion rate	No	Reference	–	–
	Yes	1.312	0.562 – 3.063	0.530
**Any complication (any Clavien Grade)**	No	Reference	–	–
	Yes	1.795	1.072 – 3.008	0.026
Minor complications (Clavien I and II)	No	Reference	–	–
	Yes	1.180	0.581 – 2.398	0.647
**Major complications (Clavien III and IV)**	No	Reference	–	–
	Yes	2.104	1.100 – 4.025	0.025
**Prostate weight (g)- specimen**	Continuous variable	1.009	1.003 – 1.015	0.006

## DISCUSSION

Hospital admission and discharge practices represent an important indicator of the quality and efficiency of medical care and have a clear impact on the healthcare costs. In an effort to minimize these costs without compromising clinical outcomes, physicians and hospital managers have increasingly focused on reducing the hospital stay following surgical procedures (12). Thus, the identification of factors that correlate with longer hospitalization time not only helps to plan expenditures, but also can help in the modification of variables that potentially increase the admission period of the patients. Our study demonstrated several interesting points regarding perioperative care, practices to minimize hospitalization and modifiable factors correlated with prolonged hospitalization.

The feasibility of promoting early hospital discharge after RRP has been demonstrated in several previous studies and different approaches have been described in order to optimize the perioperative recovery. Abou-Haidar Het al. (22) recently described a multidisciplinary approach which involves meeting with nurses prior to surgery for perioperative instructions, provision of an appropriate booklet to reinforce perioperative care, visit by an internist before and after surgery for optimization of medications, early mobilization, respiratory physiotherapy, use of PCA pump in the iPO with transition to oral medications on the first postoperative day. By adopting this standardized strategy of care, the authors were able to reduce hospital stay from a median of 3 (IQR 3 to 4 days) to a median of 2 days (IQR, 2 to 3 days, p<0.0001). The complication rates, emergency room visits and hospital readmissions were not significantly different in the pre and post-intervention groups (17% vs. 21%, p=0.80, 12% vs. 12%, p=0.95, and 3% vs. 7%, p=0.18, respectively). The perioperative care post-RRP at our institution is very similar to the one presented by these and some authors (23-25), as shown in Table-1. While hospitalization time is considered a marker of efficiency, hospital readmission is a prominent marker related to the quality of health services (26). A recent study evaluating Medicare patients, estimated that the annual cost with hospital readmissions reaches $ 17.4 billion (26-28). The impact of early hospital discharge on readmission rates has also been accessed in recent studies. Kaboli et al. (26) evaluated 4,124,907 admissions to 29 hospitals of the American Veterans Affairs (VA) system and demonstrated that the reduction in length of hospital stay does not necessarily occur at the expense of increased readmission rates; In the last 14 years, the mean length of stay in the VA system decreased by 27% (1.46 days) without any significant increase in the incidence of hospital readmission. However, in the same study, hospitals that tended to discharge earlier than expected (considering baseline disease and overall length of hospital stay in the VA system) had significantly higher rates of readmission (growth rate of 6% for each day less hospitalization than expected). It is clear, therefore, that an ideal balance between hospitalization and readmission rates should be the ultimate goal, in order to obtain the highest degree of efficiency without impairing the quality of patients’ care. To this end, the adoption of strict hospital discharge criteria respecting not only the clinical conditions, but also the logistics and patient safety are fundamental.

Direct comparisons of complication rates among different RRP series are limited due to the variations in definitions and methods of classification of surgical complications in the studies currently available in literature; additionally, very few studies evaluated the correlation between postoperative complications and hospitalization time (29). Donat et al. (30) recently analyzed the quality of the available studies evaluating complications after different urological surgeries. A total of 109 studies were identified of these, only 36 studies reported the severity of the complications and only 7 studies used some numerical classification. Furthermore, Martin et al. (31) lately proposed some strict criteria that should be followed in high quality studies reporting surgical complications including: data collection methods, duration of follow-up, outpatient information, definitions of complications, mortality and morbidity rates, specific complications rate for each procedure, graduation system and length of hospital stay. Our study is one of the rare publications on RRP outcomes available in the literature which complies with all Martin criteria (32).

We initially built a regression model including only preoperative variables; the aim of this model was to explore factors that may predict prolonged hospitalization based only on clinical parameters and tumor characteristics, which are available through medical history and clinical staging prior to the surgical procedure. Therefore, the findings of this model could be used for accurate preoperative patients counseling and could aid hospitals and healthcare payment sources in managing cost-effectiveness, hospital bed availability and healthcare resources according to the characteristics of patients awaiting RRP in each institution. In this preoperative model, we identified as independent predictors of prolonged hospital stay age, ICC (as a binary or continuous variable), ASA 3 score, TRUS prostate volume and black race. The independent predictors for prolonged hospital stay identified in our study are similar to those observed in other recent studies.

The correlation between comorbidities and age with hospitalization time after RRP has been confirmed in two recent studies. Kelly et al.(33) evaluated 2411 RRPs of the Irish Cancer Registry between 2002 and 2009. The median length of hospital stay was 8 days and in the adjusted analysis the main predictors of prolonged hospitalization were: presence of comorbidities (OR = 1.64, 95 % 1.25-2.16), advanced stage (III-IV, OR 2.19, 95% CI 1.44-3.34), and marital status single (OR = 1.71 CI 95% 125-2.34). In addition, patients submitted to treatment in high-volume hospitals (median annual PRRs> 49) or by high-volume surgeons (annual volume> 17 PRRs) had significantly lower chances of having prolonged hospitalization (OR = 0.34, 95% CI, 26-0.45, OR = 0.55, 95% CI 0.42-0.71, respectively). Co-morbidities in the study by Kelly and colleagues were assessed using the Elixhauser index (34), which includes a broad spectrum of 31 comorbidities and have demonstrated a higher discrimination power than CCI to predict inhospital mortality in previous studies (35). The presence of any of the comorbidities listed in the Elixhauser index in this study correlated with a 64% greater chance of prolonged hospitalization compared to patients without comorbidities. In our series, the presence of comorbidities (ICC≥1 vs. 0) correlated with an 82% greater chance of prolonged hospitalization; In turn, when assessing ICC as a continuous variable, we observed that each increase of 1 point in the index correlated with a 40% higher risk of prolonged hospitalization.

In turn, Trinh et al. (12) recently published an analysis of the Nationwide Inpatient Sample evaluating 89,883 RPs between 2001 and 2007. Prolonged hospitalization was defined as hospital admission greater than 3 days (75th percentile). In the multivariate analysis, the predictors of prolonged hospitalization were age (as a continuous variable, OR 1.01, 95% CI 1.01-1.02), year of surgery (2006-2007 vs. 2001-2003 OR 0.50, 95% CI 0.48-0.53), surgical volume (3 tercile vs. 1 tercile, OR 0.21, 95% CI 0.20-0.23), hospital location (West vs. Northeast, OR 0,95% CI 0.63-0.71), presence of comorbidities (ICC≥1 vs. ICC 0, OR 1.32, IC95% <0.001), surgical approach (minimally invasive vs. RRP, OR 0, 61, 95% CI 0.54-0.69), race (black versus white, OR 1.52, 95% CI 1.42-1.62), type of health insurance (Medicare versus private, OR 1, 21 95% CI, 1.16-1.28) and presence of surgical complications (OR 6.86, 95% CI 6.54-7.19). Confirming our findings, Trihn et al. (12) also observed that both CCI and age were predictors of prolonged hospitalization; each additional year of age correlated with a 1% increase in the risk of prolonged hospitalization in the Trihn et al. (12) series and with a 5% increase in our series, while the presence of comorbidities increased by 32% the risk of prolonged hospital admission in their study and by 82% in our series.

The ASA physical status classification system has been underused in the recent literature on prostate cancer. However, the importance of this classification as a predictor of complications, length of hospital stay and mortality after RRP has been demonstrated in several prior studies (36, 37). Froehner et al. (36) evaluating 444 consecutive patients with a median follow-up of 6 years demonstrated that the ASA classification is an accurate tool to improve the prognostic classification of comorbidities in RRP patients, with a greater discriminative power than the ICC in terms of overall survival and non-cancer mortality. In turn, Dillioglugil et al. (37) evaluated 472 patients treated with RRP by a single surgeon and demonstrated that ASA score of 3 correlated with a three-fold increase in the risk of complications, prolonged hospital stay, postoperative admission to ICU and blood transfusions. Major complications were also almost 3 times more frequent in ASA score 3 (21.3%) than in score 1 or 2 (7.6%) (p<0.005). Our results confirm this strong correlation between the ASA score 3 and perioperative RRP outcomes; in our logistic regression ASA 3 patients had a 3.2 times higher risk of prolonged hospitalization compared to ASA 1 patients.

Perhaps one of the most intriguing findings in our study is the correlation between black race and prolonged hospitalization; black patients had approximately a two-fold greater odds of prolonged hospitalization than white patients in our series. Similar findings were obtained by Trinh et al. (12), in their analysis, black patients presented a 52% higher chance of prolonged hospitalization compared to white patients. In our study, this result is probably correlated with variables that are potentially linked to the black race but were not included in our regression model (confounding variables). In our country, there is still a great socioeconomic disparity between white race and African-Americans, according to a recent census (38), a black worker earns, on average, just over half (57.4%) of the income received by white workers. Moreover, the data (38) shows that while 22% of the white population had completed high school in 2013, less 10% of African Americans had reached the same level of schooling. In this way, socioeconomic factors can justify the longer hospitalization obtained among black patients in our series. Unfortunately, data on income and/ or socioeconomic status were not available in our institutional database, preventing the inclusion of these variables in our regression model

When including pre, intra and post-operative variables in the regression model, CCI (continuous or binary), age, ASA score 3, black race and prostate weight maintained their statistically significant correlation with prolonged hospitalization. All variables had only small variations in their Odds Ratios, confirming, therefore, the independent correlation of these predictors with the length of hospital stay. Additionally, in this model, operative time, the presence of complications of any grade or major complications (Clavien III and IV) were identified as independent predictors for prolonged hospitalization. The impact of surgical complications on hospital stay time has been clearly established in prior series (12, 39-41), this correlation seems to be unequivocal and obvious, since patients who suffer complications require longer hospitalization time for diagnosis, treatment, and recovery from these complications. In the already cited study of Trinh et al. (12), the presence of complications was the most important predictor of prolonged hospitalization; patients who presented perioperative complications had a 7 times greater risk of prolonged hospitalization when compared to patients without complications. In the series by Chang et al. (41), perioperative complication was also an independent predictor of longer hospital stay; the rates of complication were significantly lower (p=0.013) in the group of patients who were discharged within 2 days (2.3%) in comparison with the group which stayed hospitalized for 3 days (7.0%). In our study, the presence of complications (any complication or major complications) was associated with an approximately 2 times greater odds of prolonged hospitalization, constituting one of the most significant predictors, together with an ASA score 3. Finally, the correlation between prolonged operative time, complications and prolonged hospitalization is in line with other studies. Rabbani et al. (40) evaluated 4592 consecutive patients who underwent RRP (3458) or VLRP (1134) in a single institution. In their multivariate analysis, operative time was identified as an independent predictor of surgical complications of any grade (p=0.001), together with CCI (p<0.001), BMI (p=0.01), estimated bleeding (p=0.006), and black race (p=0.027), results which are very close to the ones obtained in the present series. In our study, each additional minute of operative time was correlated with a statistically significant increase of 0.6 to 0.7% in the odds of prolonged hospitalization.

Both the complication rates and operative time can be considered as potentially modifiable variables that could reduce the risks of prolonged hospitalization. The main factors that can determine a clear decrease of both variables, with subsequent impact on length of hospital stay, are the surgeon's experience and the hospital surgical volume. It has been demonstrated that more experienced surgeons and high volume hospitals tend to present reduced complication rates, operative time, and even costs; Judge et al. (42) recently evaluated 18,027 RRPs performed between 1997 and 2004 in hospitals of the English National Health Service. The mean length of hospital stay decreased by 2.96% (95% CI, 1.98-3.92, P<0.001) per quintile of increase in hospital surgical volume, the probability of hemorrhagic complications decreased by 6% (95% CI 1-11, P=0.02) and medical complications by 10% (CI 95% 0-19, P=0.04) per quintile increase in hospital surgical volume; also re-hospitalizations within one year decreased by 15% (95% CI 6-22, P=0.001) and genitourinary complications by 5% (95% CI 2-8, P=0.002), per quintile of increase in hospital surgical volume., Finally, Coelho et al. (29) demonstrated, in a series of 2500 RRPs performed by a single surgeon, a reduction in the complication rate from 9.3% in the first 300 cases of the series to 3.3% in the last 300 cases, highlighting the concept that more experienced surgeons have less complication rates.

Our study has a number of limitations. First of all, some postoperative complications and read-missions may be underreported, especially minor complications and those managed in other hospitals. Second, our hospital is a high-volume cancer care center and, therefore, the outcomes presented herein may not be representative of general urologists in a community setting (limited external validity). Third, as the data on socioeconomic aspects were not available in our database we could not evaluate it as confounding variables in our prediction models; undoubtedly, residual confusion may explain at least some of the observed findings, since other complex and unknown factors involved in patient selection may have been left out of the regression model. Finally, it is important to highlight that CCI, used as one of the main instruments for the evaluation of comorbidities in this study, is an index initially designed to evaluate serious diseases in hospitalized patients; thus, this instrument does not address the full range of common comorbidities among patients with prostate cancer, such as hypertension, pulmonary disease and coronary artery disease (in the absence of myocardial infarction), hyperlipidemia and asthma.

Among the strengths of the study we highlight the comprehensiveness of the data collected; all patients were included in the regression models increasing its statistical power. Additionally, it is one of the few RRP series following not only the Clavien graduation system (28) but also fulfilling all the Martin criteria (31). As already pointed out, RRP series reporting complications and peri-operative morbidity through standardized methodology are fundamental for accurate patient counseling and to facilitate the comparison between different institutions and surgical approaches; such series are, however, scarce in the literature and our study adds important findings to the body of this literature. Undoubtedly, our data demonstrate realistic outcomes and expectations for patients undergoing RRP in our setting.

## CONCLUSIONS

The independent predictors of prolonged hospitalization in our series were ICC, age, ASA score 3, prostate volume on USG-TRUS, African-American race, operative time, presence of any complications and major complications. The identification of these factors allows not only better planning of institutional costs related to RRP but also proper counseling of patients undergoing RRP. Potentially modifiable risk factors, such as OR time and complications, can be optimized to shorter length of hospital stay after RRP.
